# Patchouli alcohol protects against myocardial ischaemia–reperfusion injury by regulating the Notch1/Hes1 pathway

**DOI:** 10.1080/13880209.2022.2064881

**Published:** 2022-05-19

**Authors:** Ying Lu, Shou-ye Li, Hui Lou

**Affiliations:** a Electrocardiogram room of Department of Functional Examination, Tongde Hospital of Zhejiang Province, Hangzhou, China; b College of Pharmacy, Hangzhou Medical College, Hangzhou, China

**Keywords:** Endoplasmic reticulum stress, apoptosis, cardiac function

## Abstract

**Context:**

Patchouli alcohol (PA) has protective effects on cerebral ischaemia/reperfusion (I/R) injury, but its efficacy on myocardial ischaemia-reperfusion (MI/R) has yet to be addressed.

**Objective:**

To examine the therapeutic effect of PA on myocardial ischaemia-reperfusion (I/R) injury.

**Materials and methods:**

C57BL/6 male mice were randomly divided into sham, MI/R, MI/R + PA-10, MI/R + PA-20 and MI/R + PA-40 groups. *In vivo* MI/R model was established by ligating the anterior descending coronary artery of the heart. *In vitro* stimulated IR cell model was constructed by using the rat cardiomyocyte H9C2 cell line. Mice in the treatment groups were intraperitoneally injected with PA (10, 20, 40 mg/kg) for 30 days then subjected to surgery, and cells in the experimental group were pre-treated with PA (1, 10 or 100 μmol/L). After treatment, mouse heart function, myocardial injury markers, myocardial infarction and Notch1/Hes1 expression, endoplasmic reticulum stress markers, and apoptosis-related proteins were determined.

**Results:**

*In vivo*, PA treatment improved hemodynamic parameter changes and myocardial enzymes, increased the left ventricular ejection fraction and left ventricular fractional shortening, reduced the left ventricular end-systolic diameter and inhibited CK-MB, cTnI and cTnT levels. In addition, PA attenuated myocardial tissue damage and apoptosis. PA treatment elevated Notch1, NICD and Hes1 levels and suppressed the levels of ATF4, p-PERK/PERK, and cleaved caspase-3/caspase-3 *in vitro* and *in vivo*.

**Discussion and conclusion:**

PA protects against MI/R, possibly by modulating ER stress, apoptosis and the Notch1/Hes1 signalling pathways. These findings indicate that PA may be a promising candidate for treating ischaemic heart diseases.

## Introduction

With the development of society and the ageing of the population, cardiovascular and cerebrovascular diseases have become a heavy burden on society (Pejin et al. 2012; Kivimaki 2018). Timely initiation of myocardial reperfusion thrombolytic therapy is an effective way to treat acute myocardial ischaemia injury and limit the extent of myocardial infarction. Nevertheless, this process can induce myocardial cell death, that is, myocardial ischaemia–reperfusion (MI/R) injury (Binder et al. 2015). Although much research has been conducted, the detailed molecular mechanism related to the occurrence and development of MI/R injury is still not fully understood. Consequently, there is an urgent need to understand the molecular mechanisms behind MI/R damage to find potential MI/R treatments.

*Pogostemon cablin* Benth., also known as patchouli, or ‘Guanghuoxiang’ in traditional Chinese medicine, is a member of the Lamiaceae family of flowering plants with anti-inflammatory, antimicrobial, antitumor and antioxidant effects (Chen et al. 2021). Current research on patchouli mainly focuses on its volatile oil, namely, patchouli oil (PO). As the essential oil obtained from the dry leaves of *Pogostemon cablin*, PO has been reported to have several pharmacological activities including antioxidation, anti-inflammation, immunomodulatory, antinociceptive and antiallergy effects (Zhang et al. 2016; Gan et al. 2020). Patchouli alcohol (PA) is the most abundant PO and has received widespread attention (Junren et al. 2021). PA has various biological activities, including anti-inflammatory, antioxidant, metabolic regulation, etc. (Lee et al. 2020). Wei et al. (2018) reported that PA exerts anti-inflammatory effects to reduce brain I/R damage in obese mice. However, whether PA has a similar protective effect on MI/R is unclear.

The Notch signalling pathway is related to ischaemia–reperfusion (I/R) injury. Studies have found that activation of Notch1 can reduce brain I/R injury, MI/R injury, and intestinal I/R injury (Chen G et al. 2013; Jin et al. 2019; Xu et al. 2021). Hairy and enhancer of split (Hes) is a key effector molecule in the Notch signalling pathway. The Notch1/Hes1 signalling pathway protects cardiomyocytes from I/R damage (Zhang M et al. 2017). The endoplasmic reticulum (ER) can maintain the normal physiological functions of cells under normal conditions, but the function of the ER under ischaemic conditions is interfered with, which will cause ER stress (Glembotski 2007; Wang et al. 2016). ER stress can activate downstream apoptosis signalling pathways and cause cardiomyocyte apoptosis (Wang et al. 2016). ER stress activates the PERK/ATF4 signalling pathway, thereby activating the apoptosis signalling pathway in I/R injury (Szegezdi et al. 2006). Therefore, activating the Notch1/Hes1 signalling pathway and inhibiting the PERK/ATF4 pathway have become strategies to reduce MI/R damage.

PA can protect against cerebral I/R injury, but its efficacy in MI/R has yet to be investigated. Therefore, in the current study, we performed *in vivo* and *in vitro* experiments to study the protective role of PA against MI/R injury.

## Materials and methods

### PA preparation

PA (IP0680, purity ≥ 98%) was provided by Solarbio Life Science, Beijing, China. For *in vitro* assays, PA was dissolved and diluted with dimethyl sulfoxide (DMSO) to prepare final concentrations of 0, 0.1, 1, 10, 100 and 1000 μmol/L PA. For *in vivo* assays, PA was dissolved in saline containing 5% DMSO at concentrations of 10, 20 and 40 mg/kg.

### Animals

Thirty C57BL/6 male mice (6–8 weeks old, 20–22 g) were provided by Shanghai Slake Animal Laboratory Co. Ltd. (Certificate No. SCXK (Hu) 2017-0005, Shanghai, China). The mice were housed under specific pathogen-free conditions and given free access to food and water. The room temperature was maintained at 20 °C ± 2 °C. Our research was approved by the Ethics Committee of Zhejiang Traditional Chinese Medicine University (Hangzhou, China; approval number ZSLL-2019-036).

### MI/R mouse model and treatment

The mice were randomly divided into five groups (*n* = 6 per group): the sham group, MI/R group, MI/R + PA-10 group, MI/R + PA-20 group and MI/R + PA-40 group. The MI/R model was established according to the research of Zhang et al. (2017). Briefly, after anaesthetisation with 1% sodium pentobarbital, the chest wall of the mice was cut open, and the left anterior descending coronary artery (LAD) was ligated with 6-0 silk thread. After 30 min of ischaemia, the slip knot was released for reperfusion injury. Mice in the sham operation group did not have LAD ligation, and the other procedures were the same as those in the MI/R group. The mice in the treatment groups were intraperitoneally injected with PA (10, 20 and 40 mg/kg) for 30 days before surgery.

### Echocardiography

Mice were anaesthetized with 1.5% isoflurane 72 h after the operation. Then, the ultrasound coupling agent was applied to the chest of mice, and the probe was closely attached to the chest of mice. The heart function of mice was detected by the VEVO 770 high-resolution imaging system (VisualSonics, Canada). Left ventricular end-systolic diameter (LVESD) and left ventricular end-diastolic diameter (LVEDD) were recorded by two-dimensional M-mode echocardiography. Left ventricular ejection fraction (LVEF) and left ventricular fractional shortening (LVFS) were calculated as previously reported (Yu et al. 2018b).

### Infarct size measurement

Triphenyl tetrazolium chloride (TTC) staining was used to determine the infarct size after MI/R injury. After MI/R, the mouse hearts were excised, frozen, and cut into 2 mm slices. TTC (1% Sigma–Aldrich) was used to stain the tissue sections, which were incubated for 5 min at 37 °C and fixed with 4% paraformaldehyde to detect the myocardial infarction. The white regions indicate myocardial infarction size; red areas represent non-ischaemic regions. Finally, the infarct size was calculated using Image Pro Plus software.

### Enzyme-linked immunosorbent assay (ELISA)

After echocardiography, the mice were anaesthetized with pentobarbital sodium, and blood was taken from the main vein of the abdomen. After centrifugation, the upper serum was separated for ELISA determination. ELISA kits for creatine kinase MB (CK-MB, MM-43703M2), cardiac troponin (cTnI, MM-0791M2) and cardiac troponin T (cTnT, MM-0945M2) were purchased from MEIMIAN (http://www.mmbio.cn/). The operation steps were carried out following the instructions. In brief, samples and standards were added to the 96-well plate and then reacted with the enzyme-labeled reagent for 30 min. Then, a colour-developing solution was used to develop the colour, and a stop solution was used to terminate the reaction. Finally, the corresponding optical density (OD) value was read through a microplate reader (TECAN, Mannedorf, Switzerland).

### Haematoxylin and eosin staining

After the blood was taken, the mouse heart was quickly separated, and one-third to two-thirds of the apex of the heart was taken for paraffin sectioning. After the sections were deparaffinized and hydrated, they were stained with haematoxylin (G1142, Solarbio, China) and eosin (G1100, Solarbio, China). Afterward, the sections were dehydrated, cleared, and sealed with neutral balsam mounting medium (E675007, Sangon Biotech, China). The degree of damage to the mouse myocardial tissue was observed under a microscope (BX53 M, Olympus, Japan).

### Western blot

One-third of the mouse heart apex was used for western blot detection. Protein lysis and extraction were performed with RIPA lysis buffer (R0010, Solarbio, China), and the quantification of extracted protein was determined with a BCA protein kit (PC0020, Solarbio, China). Afterward, protein lysates were electrophoresed with SDS-PAGE and transferred to a polyvinylidene fluoride membrane, which was blocked with BSA (5%, SW3015, Solarbio, China) for 2 h and incubated with primary antibodies and secondary antibodies. The visualisation of membranes was performed by an ECL kit (PE0010, Solarbio, China) on an iBright CL750 Imaging System (A44116, Thermo Fisher Scientific, USA).

The antibodies purchased from Abcam (UK) used in this study were as follows: anti-Notch1 antibody (ab52627), anti-Hes1 antibody (ab108937), anti-ATF4 antibody (ab216839), anti-PERK antibody (ab229912), anti-phospho-PERK antibody (ab192591), anti-caspase-3 antibody (ab184787), anti-cleaved caspase-3 antibody (ab214430), anti-β-actin antibody (ab8226), goat anti-rabbit (ab205718), goat anti-mouse (ab6789) and anti-NICD antibody (2423S, CST). β-Actin was employed as an internal control.

### Cardiomyocyte culture

H9C2 rat cardiomyocytes (CL-0089, Procell, Wuhan, China) were cultured in Dulbecco’s modified Eagle’s medium (DMEM, D5030, Sigma–Aldrich, USA) supplemented with 10% foetal bovine serum (F2442, Sigma-Aldrich, USA) and 100 U/mL–100 μg/mL penicillin–streptomycin (P4333, Sigma-Aldrich, USA) at 37 °C and 5% CO_2_. Stimulated IR (SIR) treatment was carried out using physiological concentrations of potassium, hydrogen, and lactate as described previously.

### Simulated ischaemia/reperfusion (SI/R) model and treatment

The experiments were divided into five groups: control group (normally cultured), simulated ischaemia/reperfusion (SI/R) group, SI/R + PA-1 group, SI/R + PA-10 group and SI/R + PA-100 group. The last three groups of cells were treated with PA (1, 10 and 100 μmol/L) for 8 h before SI/R treatment in serum-free medium. The SI/R group was subjected to SIR treatment in serum-free medium. SIR treatment was carried out using physiological concentrations of potassium, hydrogen and lactate as described previously (Yang et al. 2013). Briefly, H9C2 cells were cultured with ischaemic buffer containing 4 mM HEPES, 0.9 mM CaCl_2_·2H_2_O, 0.49 mM MgCl_2_, 137 mM NaCl, 12 mM KCl, 10 mM deoxyglucose, 0.75 mM Na_2_SO_3_ and 20 mM C_3_H_5_O_3_Na (pH = 6.5) in a Galaxy® 48 R hypoxia incubator (CO48200005, Eppendorf AG, Germany) with 5% CO_2_ and 95% N_2_ for 2 h. Afterward, the cells were washed with phosphate-buffered saline (PBS) and cultured in an oxygen chamber with 5% CO_2_ and 95% atmosphere for 4 h.

### CCK-8 assay

H9C2 cells were treated with 0, 0.1, 1, 10, 100 and 1000 μmol/L PA for 8 h, in the presence or absence of SI/R treatment and then cultured in 96-well plates at 37 °C with 5% CO_2_. Then, 10 μL CCK-8 reagent (HY-K0301, MedChemExpress, USA) was added to the plates and incubated at 37 °C for 4 h. The OD value at 450 nm absorbance was recorded and determined using a microplate reader.

### TUNEL staining

TUNEL staining of myocardial tissue was carried out with the help of the TUNEL Apoptosis Detection Kit (C1091, Beyotime, China). After the sections were deparaffinized and hydrated, they were incubated with proteinase K (ST532, Beyotime, China) for 30 min. Afterward, the sections were washed and reacted with 3% hydrogen peroxide solution at room temperature for 20 min. The biotin labelling solution was added to the sections and incubated for 1 h. After washing with PBS, a freshly prepared streptavidin-HRP working solution was added to the sections, followed by incubation at room temperature for 30 min. DAB solution was used for colour development, and haematoxylin staining solution was used for nuclear staining. The results were observed under a microscope. The apoptotic index was calculated as the ratio of the number of apoptotic cells (positive TUNEL stained cells) to the total number of cells.

### Statistical analysis

Every experiment was performed more than three times in an independent manner. Data are expressed as the mean ± standard deviation (SD). Statistics were analysed using GraphPad 8.0 software (GraphPad, Inc., La Jolla, CA, USA). Statistical significance was determined by one-way ANOVA followed by a Tukey *post hoc* test and an independent *t*-test. The *p* < 0.05 was considered statistically significant.

## Results

### PA treatment relieved myocardial dysfunction in MI/R mice

**Figure 1.**
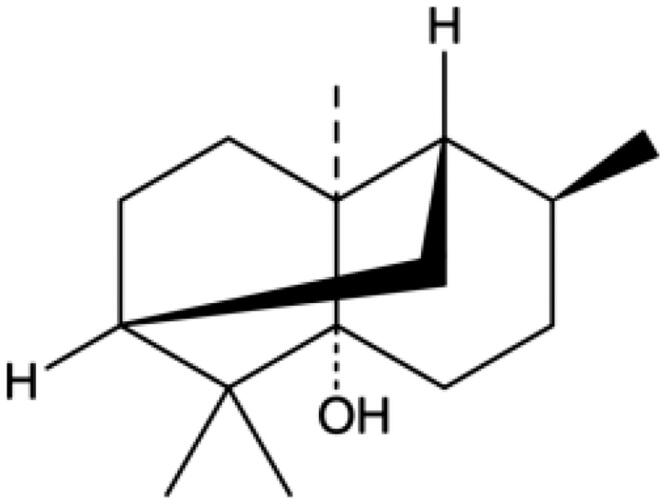
The molecular structure of PA: C_15_H_26_O.

**Figure 2.**
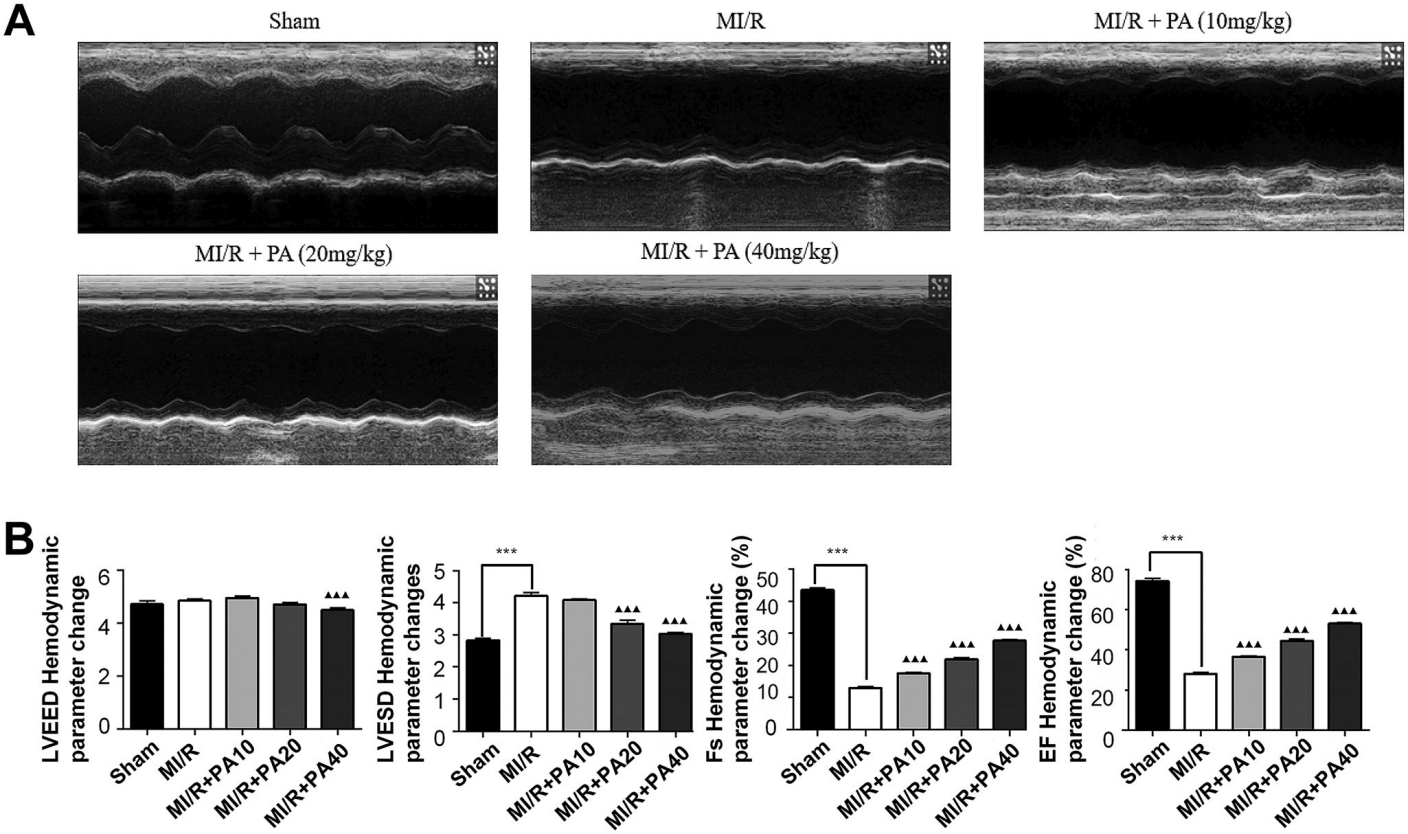
The effect of PA on cardiac function in MI/R injured hearts. (A) Cardiac function was assessed by echocardiography 48 h after MI/R operation. (B) Hemodynamic parameter changes in LVESD, LVEED, LVEF and LVFS were detected. Data are expressed as the mean ± SD. ***p < 0.001 vs. sham group. ^▲▲▲^*p* < 0.001 vs. MI/R group. LVEDD: left ventricular end diastolic diameter; LVEF: left ventricular ejection fraction; LVESD: left ventricular end systolic diameter; LVFS: left ventricular fractional shortening.

**Figure 3.**
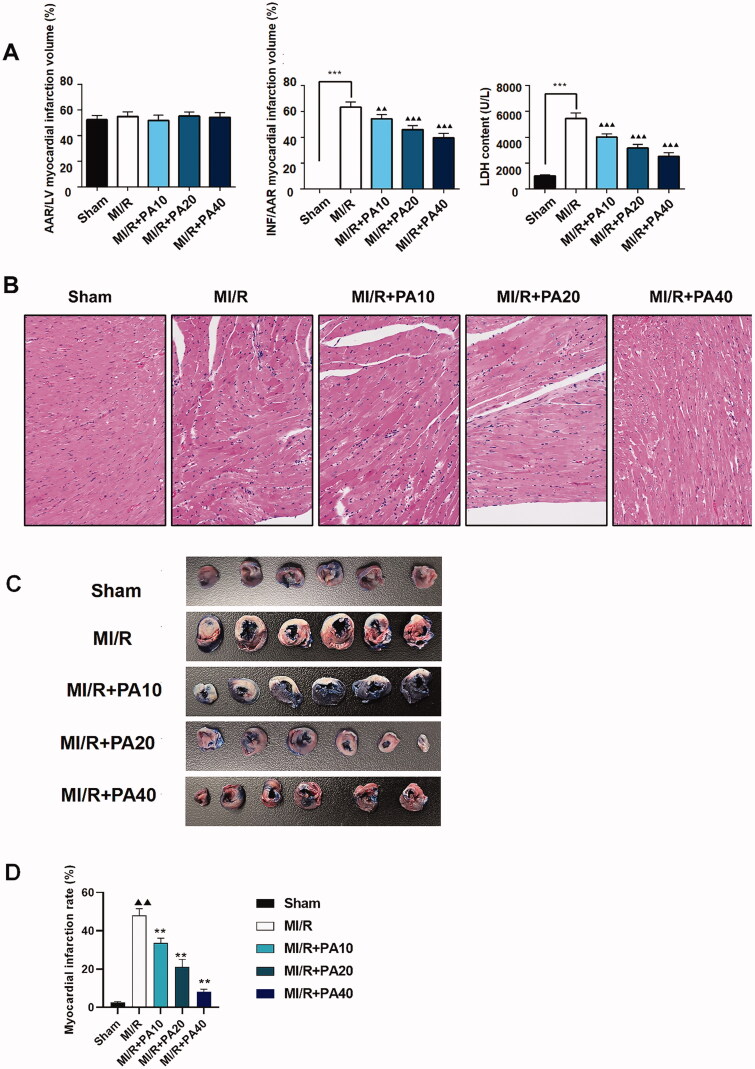
The effect of PA treatment on myocardial infarction and myocardial injury in MI/R mice. (A) Calculation of myocardial infarction volume (AAR/LV, INF/AAR) and LDH content. (B) Myocardial tissue was stained with H&E. ***p < 0.001 vs. sham group. ^▲▲^*p* < 0.01, ^▲▲▲^*p* < 0.001 vs. MI/R group. Data are expressed as the mean ± SD. (C,D) TTC staining was used to determine the mouse myocardial infarction rate in the indicated groups. ^▲▲^*p* < 0.01 vs. sham group. **p < 0.01 vs. MI/R group.

The chemical structure of PA is shown in Figure 1. M-mode echocardiography was used to evaluate cardiac parameters and the effect of PA on mouse cardiac function. We detected the left ventricular function of MI/R mice with or without PA treatment (Figure 2(a)). As shown in Figure 2(b), the LVESD of the MI/R mice increased, while the FS and EF decreased significantly, compared with the sham group (*p* < 0.001). However, PA treatment (10, 20 or 40 mg/kg) decreased the LVESD and increased the FS and EF of MI/R mice in a concentration-dependent manner (*p* < 0.05). PA did not affect the ischaemic area (AAR/LV) of the heart but alleviated the infarct size (INF/AAR) and decreased the expression of the apoptotic factor lactate dehydrogenase (LDH; Figure 3(a)). We observed damage to the mouse myocardial tissue by H&E staining. Apoptosis and inflammatory cell infiltration of myocardial cells in PA-treated mice were significantly lower than those in MI/R mice (Figure 3(b)). Moreover, the myocardial infarction size was determined by TTC staining. The results showed that the mouse myocardial infarction rate was significantly elevated in the MI/R group compared with the sham group, while PA treatment significantly reversed the increased myocardial infarction in the MI/R group in a dose-dependent manner (Figure 3(c,d)).

**Figure 4.**
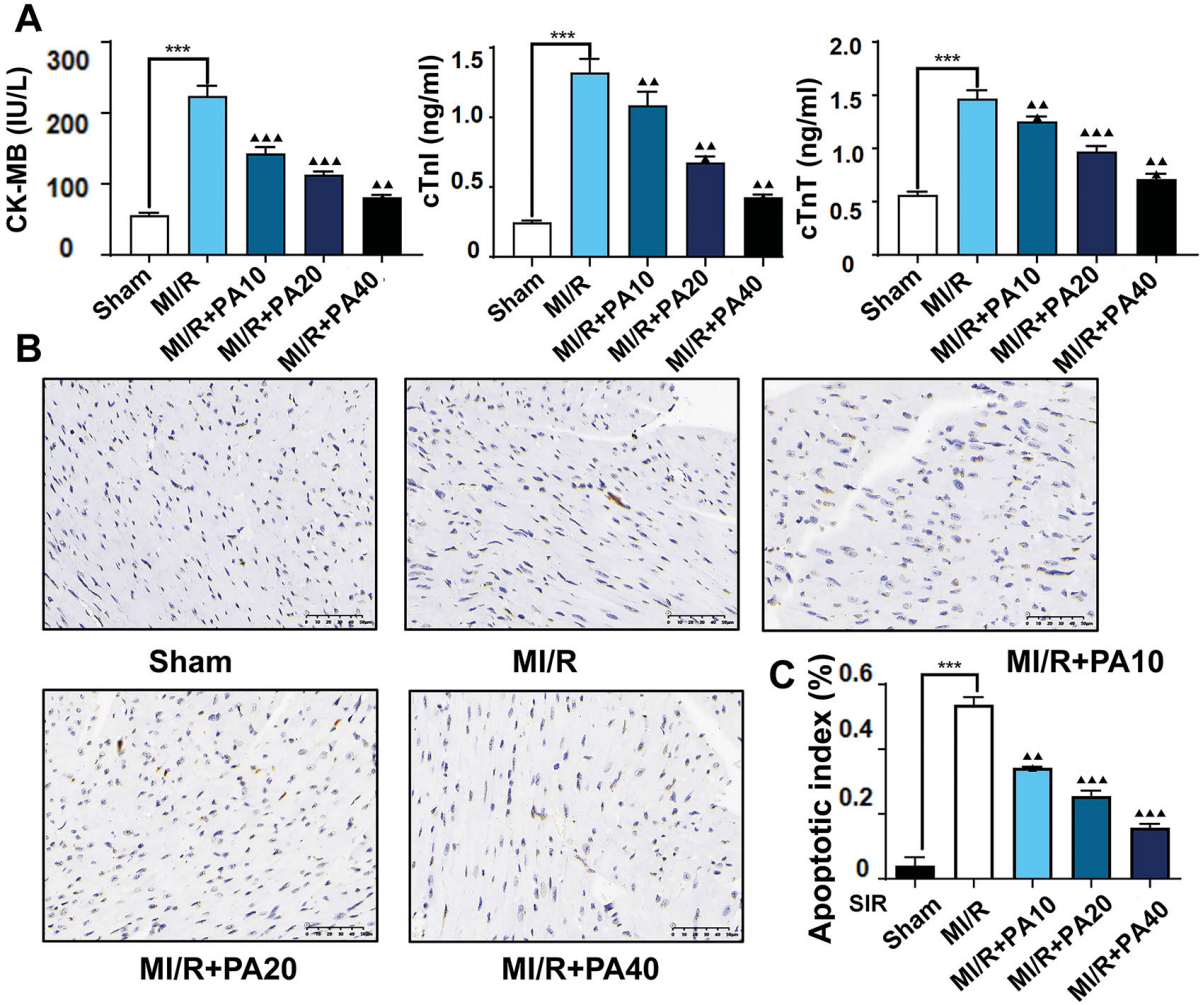
The effect of PA treatment on myocardial infarction and myocardial apoptosis in MI/R mice. (A) Concentrations of myocardial infarction factors (CK-MB, cTnI and cTnT) were detected by ELISA. (B,C) A TUNEL assay was used to evaluate the effect of PA on apoptosis in myocardial tissue. ***p < 0.001 vs. sham group. ^▲▲^*p* < 0.01, ^▲▲▲^*p* < 0.001 vs. MI/R group. Data are expressed as the mean ± SD.

We also measured myocardial infarction-related factors in mouse serum by ELISA and found that PA dose-dependently reversed the increase in CK-MB, cTnI and cTnT levels caused by MI/R (Figure 4(a)). In addition, TUNEL analysis revealed that the apoptotic rate of myocardial tissue in MI/R mice increased significantly, but this phenomenon was dose-dependently reversed by PA treatment (Figure 4(b,c)).

### PA treatment regulated ER stress and the Notch1/Hes1 signalling pathway in MI/R mice

**Figure 5.**
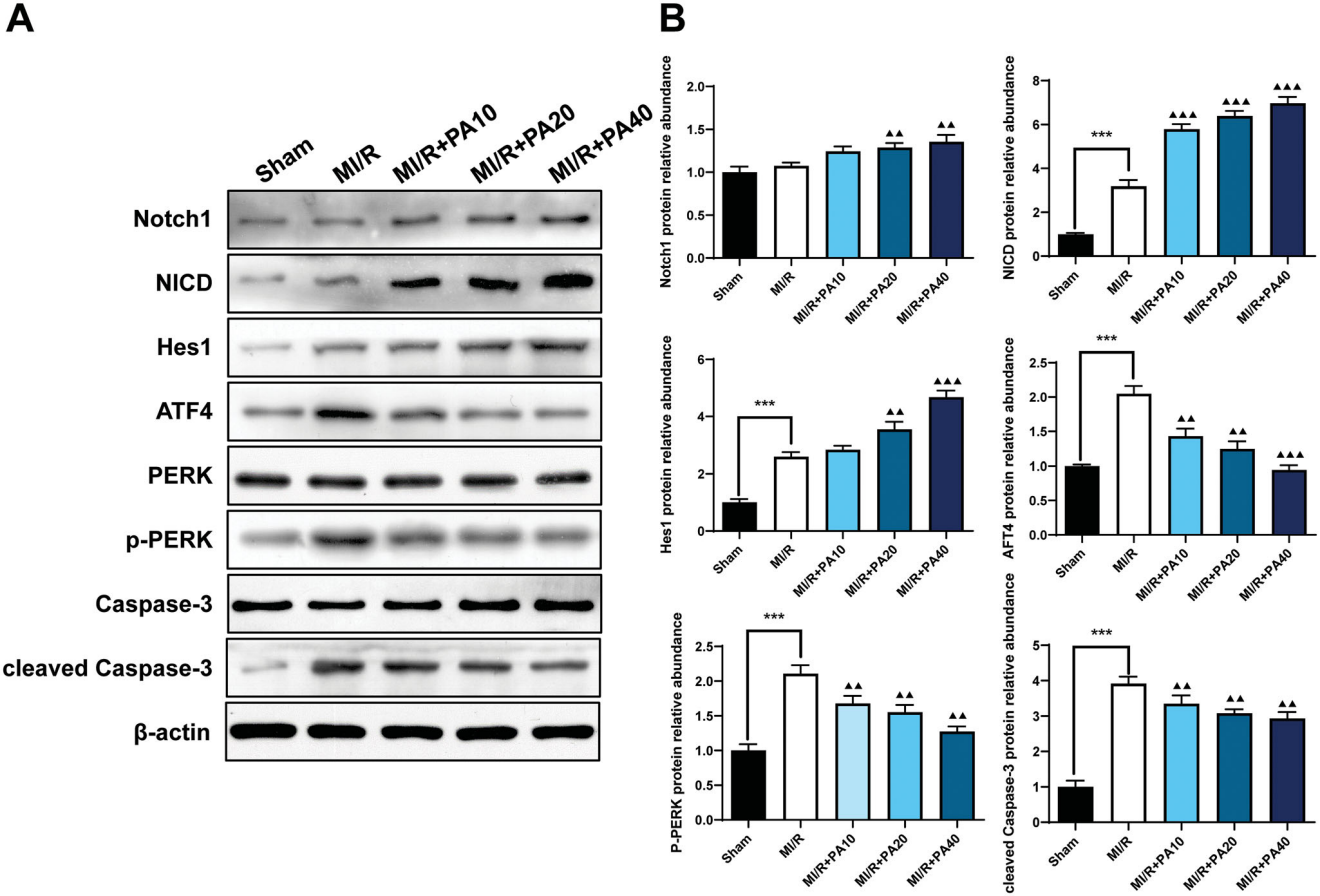
The effect of PA treatment on the Notch1/Hes1 signalling pathway and ER stress in MI/R mice. Western blotting was used to detect the levels of Notch1, NICD, Hes1, ATF4, PERK, p-PERK, caspase-3 and p-caspase-3 in MI/R mouse myocardial tissue. (A) Western blot bands of the detected proteins. (B) Relative expression levels of the proteins. ***p < 0.001 vs. sham group. ^▲▲^*p* < 0.01, ^▲▲▲^*p* < 0.001 vs. MI/R group. Data are expressed as the mean ± SD.

The Notch signalling pathway is related to ER stress and cardiomyocyte apoptosis caused by MI/R (Zhang et al. 2017). As a result, we used western blotting to detect proteins related to Notch1/Hes1 signalling as well as the expression of ER stress- and apoptosis-related proteins. Compared to the sham group, the MI/R group had significantly increased levels of NICD, Hes1, ATF4, p-PERK and cleaved caspase-3; however, the levels of Notch1, PERK and caspase-3 did not change significantly. PA treatment increased the expression levels of Notch1, NICD and Hes1 in a concentration-dependent manner. Furthermore, compared to MI/R, PA treatment significantly reduced the expression levels of ATF4, p-PERK and cleaved caspase-3 (Figure 5(a,b)).

### PA treatment increased viability, inhibited ER stress markers and activated the Notch1/Hes1 pathway in S/IR-treated H9C2 cells

**Figure 6.**
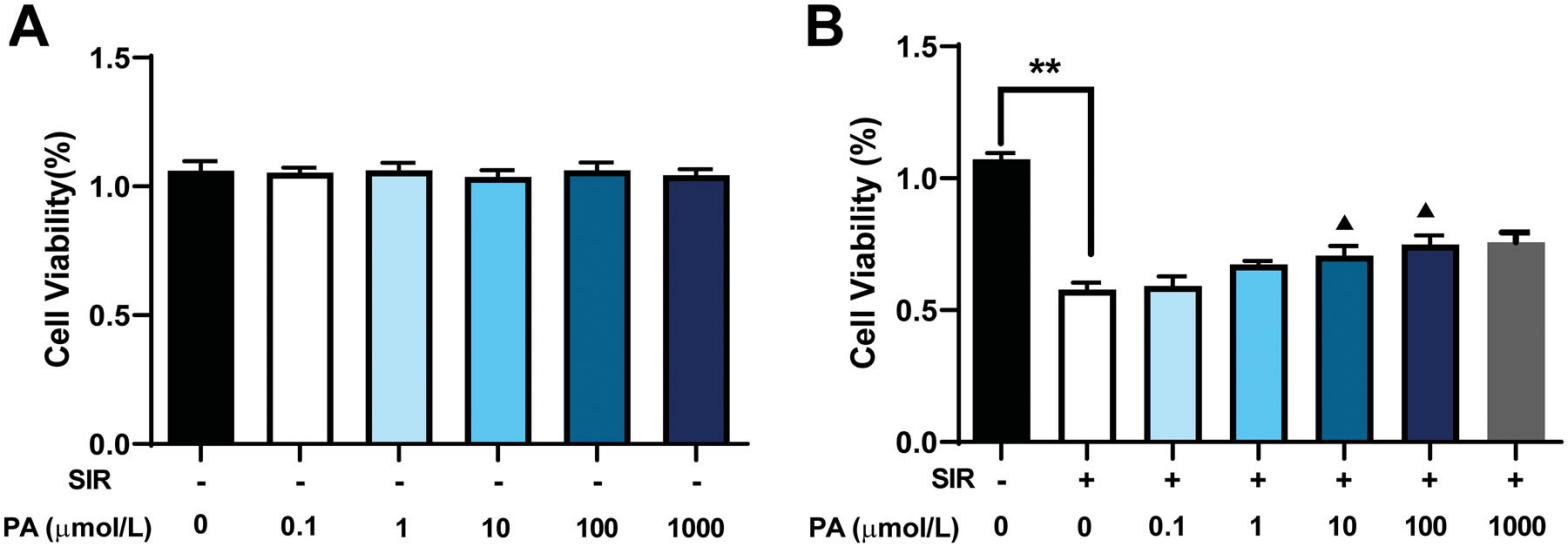
The effect of PA treatment on cell viability and apoptosis in SIR-treated H9C2 cells. (A) The effect of different concentrations of PA on H9C2 cell viability was determined by CCK-8 assay. (B) The effect of different concentrations of PA on SIR-treated H9C2 cell viability was determined by CCK-8 assay. **p < 0.01 vs. the control group. ^▲^*p* < 0.05 ^▲▲^*p* < 0.01 vs. the SIR group. Data are expressed as the mean ± SD.

To determine whether PA could protect against cardiac injury *in vitro*, we first determined its effect on the viability of H9C2 cells using CCK-8. H9c2 cardiomyoblasts were exposed to various concentrations of PA (0, 0.1, 1, 10, 100 and 1000 μmol/L) for 8 h. As shown in Figure 6(a), no significant difference in cell viability was observed when compared to the sham group. The cells were then treated with SIR in the absence or presence of PA (0.1, 1, 10, 100 or 1000 μmol/L). SIR treatment significantly decreased cell viability compared with that of the control group, while PA (1, 10 or 100 μmol/L) treatment significantly increased cell viability in a dose-dependent manner (Figure 6(b)). However, 0.1 μmol/L PA exhibited almost no effect on improving cell viability compared with the group treated with SIR alone; the effect of 1000 μmol/L PA on improving cell viability was not better than the effect of 100 μmol/L PA.

**Figure 7.**
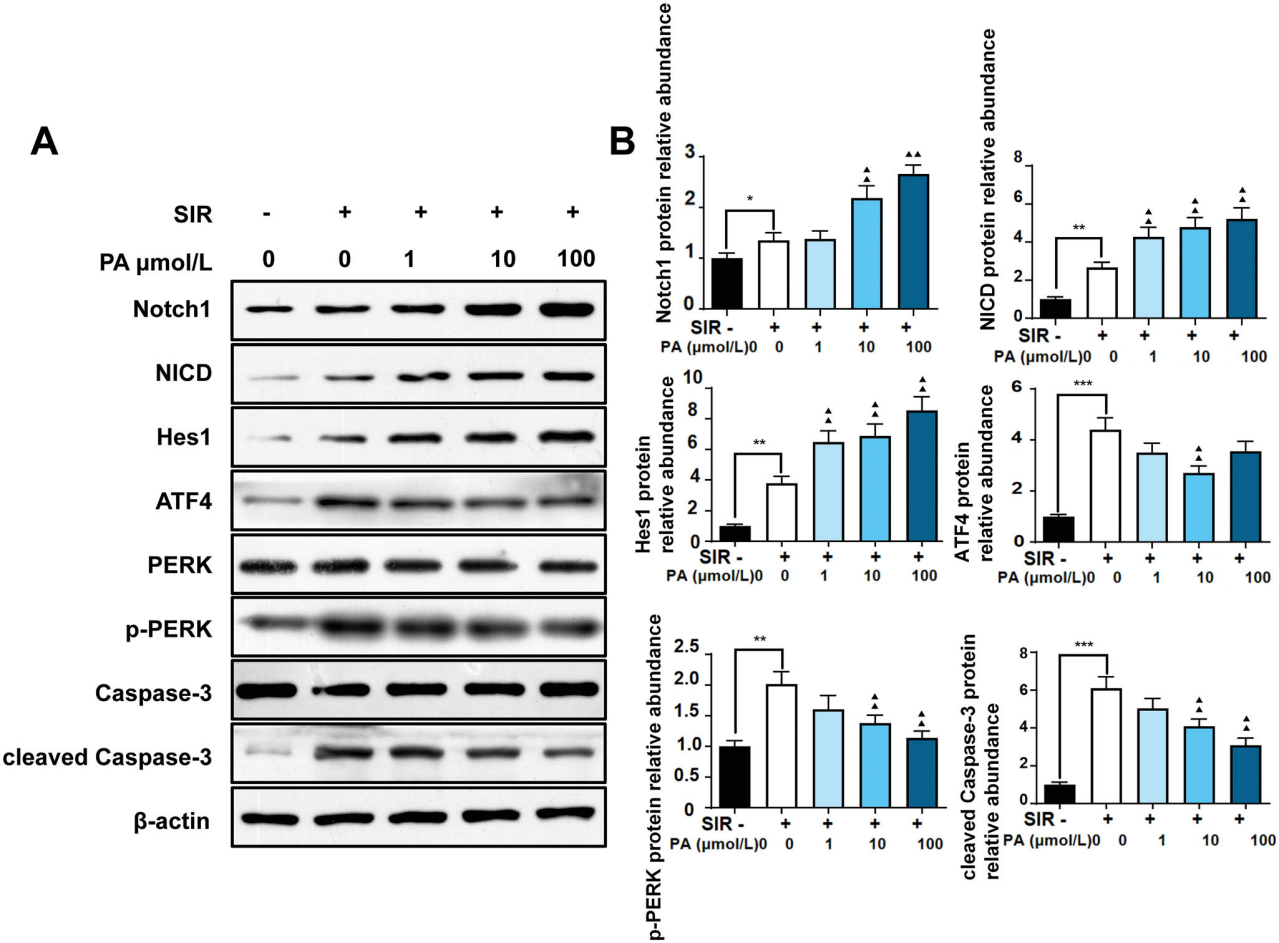
The effect of PA treatment on the Notch1/Hes1 signalling pathway and ER stress in H9C2 cells. Western blotting was used to detect the effect of PA on the contents of Notch1, NICD, Hes1, ATF4, PERK, p-PERK, caspase-3 and p-caspase-3 in SIR-treated H9C2 cells. (A) Western blot bands of the detected proteins. (B) Relative expression levels of the proteins. **p < 0.01, vs. control group. ^▲^*p* < 0.05, ^▲▲^*p* < 0.01 vs. the SIR group. Data are expressed as the mean ± SD.

Consistent with the results of *in vivo* experiments, PA treatment facilitated the expression of Notch1, NICD and Hes1 but suppressed the expression of ATF4, p-PERK, and cleaved caspase-3 in SIR-treated H9C2 cells (Figure 7(a,b)), indicating that PA activated the Notch signalling pathway and suppressed ER stress and apoptosis in both cell I/R models and animal I/R models.

## Discussion

MI/R damages the heart in many ways, including the inhibition of heart function, resulting in the abnormal energy metabolism of myocardial tissue and enlargement of the myocardial infarction area. In this study, we evaluated the protective effect of PA on MI/R injury from multiple perspectives. Cardiac ultrasound detection revealed that myocardial dysfunction in MI/R mice was relieved after PA treatment in a dose-dependent manner. Studies have shown that the cardiac function indexes LVEF and LVFS of I/R mouse models are lower than those of the control group, while LVEDD and LVESD are higher than those of the control group (Yu et al. 2018b; Wu et al. 2020). In our study, we detected these parameters related to cardiac function in MI/R mice and found similar changes in MI/R mice. However, after PA treatment, the abnormal LVER, LVFS and LVESD changes in MI/R mice were reversed. Moreover, previous studies have reported that high levels of cTnI, cTnT, CK-MB and LDH are detected in patients with acute myocardial infarction (Lim et al. 2019). In addition, increased expression of myocardial injury markers has been observed in I/R rats (Huang et al. 2020). Thus, the levels of specific cardiac biomarkers in MI/R mouse tissues were also detected in this study. We found that MI/R mice also showed high cTnI, cTnT, CK-MB and LDH levels, which were reversed by PA treatment in a dose-dependent manner. PA was indicated to exert a protective effect against myocardial infarction by regulating of myocardial injury markers.

In addition, we also evaluated myocardial tissues of MI/R mice using H&E staining and TUNEL staining. A previous study pointed out that PA prevents brain I/R damage by inhibiting the secretion of inflammatory factors (Wei et al. 2018). PA has also been revealed to have a protective effect on gastric epithelial cell apoptosis induced by oxidative stress (Xie et al. 2016, 2020). This finding indicates that the inhibitory effect of PA on myocardial tissue apoptosis may be achieved by inhibiting oxidative stress. In our study, we found that PA treatment reduced myocardial cell necrosis, apoptosis and inflammatory cell infiltration in MI/R mice. PA treatment not only had a protective effect on MI/R mouse models but was also effective for SIR cell models. A previous study observed that the viability of H9C2 cells treated with hydrogen peroxide was significantly lower than that of the control group (Zhao et al. 2021). The SIR cell model established in this study showed similar decreased viability of H9C2 cells, while after PA treatment, H9C2 cells after SIR treatment showed an increase in cell viability, and the PA concentration of 1, 10 or 100 μmol/L showed significance in elevating the cell viability. Moreover, we found that PA treatment alone did not significantly influence the viability of H9C2 cells without SIR treatment. The results indicated the potential of PA in the treatment of myocardial injury.

Oxidative stress, inflammation, and hypoxia can disrupt the homeostasis of the ER and lead to ER stress, an important signalling event in which MI/R causes tissue damage. A study found that the entry of reactive oxygen species into the ER can activate PERK, thereby phosphorylating the substrate EIF2α and losing the ability to initiate protein translation (Verfaillie et al. 2012). The activation of PERK increases the expression of ATF4 and further enhances cell damage induced by ER stress (Tao et al. 2019). Wu et al. (2019) reported that PA could protect against liver steatosis by reducing ER stress. Yu et al. (2016) demonstrated that by reducing PERK/ATF4-mediated ER stress, MI/R damage was attenuated. Therefore, we hypothesized that PA relieved MI/R injury by inhibiting ER stress. Consistent with the reported research, we found that the protein expression of ATF4, p-PERK and cleaved caspase-3 was elevated in the MI/R mouse and cell models, which was significantly reversed by PA treatment in a dose-dependent manner.

PA suppressed the activation of PERK/ATF4 *in vivo* and *in vitro* MI/R models, thereby reducing apoptosis caused by ER stress. We also evaluated the regulatory effect of PA on the Notch1/Hes1 signalling pathway, which is related to I/R injury. Yu et al. (2018a) found that polydatin increased the expression of Notch1 and Hes1, thereby reducing myocardial damage in MI/R. In addition, they also found that melatonin can prevent MI/R by activating the Notch1/Hes1 pathway (Yu et al. 2015). Consistent with previous research, in our study, we found that PA treatment dose-dependently increased the levels of Notch1, NICD and Hes1 in MI/R mouse tissues and H9C2 cells after SIR treatment. These results indicated that PA inhibited cardiomyocyte apoptosis and improved cardiac function by activating the Notch1/Hes1 signalling pathway. However, there are also some limitations to our current study that require further exploration. The possible interaction between Notch1/Hes1 and the PERK/ATF4 pathway has not been elucidated. Second, few studies have focussed the effect of PA on myocardial injury, and it is of great necessity to perform clinical investigation.

## Conclusions

The current research reveals that PA protects against MI/R *in vivo* and *in vitro*, possibly by regulating the Notch1/Hes1 and PERK/ATF4 pathways. PA therefore constitutes a potential drug for MI/R treatment. Further research into whether PA alleviates MI/R by regulating other mechanisms, such as myocardial mitochondrial dynamics, is needed to better understand the complicated pathogenesis of MI/R.

## Data Availability

The corresponding author can provide the data that support the findings of this study upon request.
